# Cancer Treatment Disruption by Residence Region in the Aftermath of Hurricanes Irma and María in Puerto Rico

**DOI:** 10.3390/ijerph21101334

**Published:** 2024-10-08

**Authors:** Francisco Muñoz-Torres, Marievelisse Soto-Salgado, Karen J. Ortiz-Ortiz, Xavier S. López-León, Yara Sánchez-Cabrera, Vivian Colón-López

**Affiliations:** 1 Division of Cancer Control and Population Sciences, University of Puerto Rico Comprehensive Cancer Center, San Juan, PR 00936, USA; francisco.munoz@upr.edu (F.M.-T.); marievelisse.soto1@upr.edu (M.S.-S.); karen.ortiz@upr.edu (K.J.O.-O.); yara.sanchez@upr.edu (Y.S.-C.); 2 Department of Health Services Research, Graduate School of Public Health, University of Puerto Rico, Medical Sciences Campus, San Juan, PR 00936, USA; 3 Office of Community Outreach and Engagement, University of Puerto Rico Comprehensive Cancer Center, San Juan, PR 00936, USA; xavier.lopez6@upr.edu

**Keywords:** cancer patients, treatment disruption, healthcare access, Hispanics, disasters, Puerto Rico, hurricanes, regional disparities

## Abstract

Since 2017, Puerto Rico has faced environmental, economic, and political crises, leading to the emigration of healthcare workers and weakening the healthcare system. These challenges have affected cancer treatment continuity, exacerbating healthcare access challenges island-wide. In this study, we estimate the effect of the residence region on cancer treatment disruption following Hurricanes Irma and María (2017). Telephone surveys were conducted with 241 breast and colorectal cancer patients aged 40 and older who were diagnosed within six months before the hurricanes and were receiving treatment at the time of the hurricanes. Treatment disruption was defined as any pause in surgery, chemotherapy, radiotherapy, or oral treatment due to the hurricanes. Prevalence ratios (PRs) of treatment disruption by residence region were estimated using the San Juan Metropolitan Area (SJMA) as the reference. Fifty-nine percent of respondents reported treatment disruption; among them, half experienced disruptions lasting more than 30 days, with 14% of these enduring disruptions longer than 90 days. Adjusted models showed a 48% higher prevalence of disruption outside the SJMA (PR = 1.48, 95% CI: 1.06–2.07). Specific geographic regions (Arecibo, Bayamón, Caguas, and Mayagüez) exhibited higher disruption prevalence. These findings emphasize the need for disaster preparedness strategies that ensure equitable healthcare access for all cancer patients following environmental calamities.

## 1. Background

In 2017, Hurricanes Irma and María wreaked havoc in Puerto Rico, resulting in widespread devastation, substantial resource depletion, and profound economic consequences. Puerto Rico’s fragile healthcare system collapsed after the hurricanes, resulting in an estimated 4645 (95% CI: 793, 8498) excess deaths attributable to the hurricanes, primarily due to the disruption of life-sustaining treatments caused by the longest blackout in U.S.A history [1,2]. This loss of human life compounded the extensive infrastructure and economic damage inflicted by the disaster. The migration of healthcare professionals from Puerto Rico following the environmental calamities led to significant shortages in healthcare personnel [2]. The resulting shortages further deepened inequities in healthcare access and, consequently, in morbidity and mortality [3,4,5]. 

Hurricanes can severely impact cancer treatment and survivorship care by causing extended power outages, damaging hospitals and specialized therapy equipment, contaminating water supplies, disrupting transportation, and displacing patients and medical staff. These disruptions hinder the availability and effectiveness of essential medical services, leading to significant challenges in maintaining continuous cancer care [6]. Compromised infrastructure, communication systems, medication access, and medical records contribute to delays in cancer diagnosis and treatment, amplifying preexisting disparities in morbidity and mortality [7,8,9]. Hurricane Katrina caused similar disruptions in 2005 in Louisiana, where individuals with chronic diseases faced significant treatment disruptions. Lack of access to physicians, medication, and financial or health insurance problems were the most common reasons for disrupted medical treatment [10]. In addition to the barriers that many oncology patients already face, limited transportation and the availability of screening facilities further compound these challenges [11,12]. Areas far from healthcare facilities and communities with socioeconomic challenges are impacted in particular [13,14]. In Puerto Rico, oncologists are concentrated in more densely populated cities, with little or no representation in less populated and more rural municipalities [15]. Similar disparities in access to cancer care providers by region have been observed in other countries, indicating that geographic location can increase delays in cancer treatment initiation and continuation [16,17,18].

In Puerto Rico, the concept of “residence region” encompasses a variety of geographic and logistical factors that significantly influence healthcare access. The regionalization of services, conceptualized in 1958, aimed to guarantee access to healthcare services at different levels, from primary care in each municipality to secondary and tertiary care at the regional level. Health regions were divided into five regions based on population count, municipality geographic size and proximity, epidemiological characteristics, and political delimitations. During the 1970s, health regions were restructured into seven regions with the purpose of improving the operation of the public healthcare system [19]. The Health Resource and Service Administration (HRSA) classified 92.3% of Puerto Rico’s municipalities as medically underserved. The remaining healthcare workers available in Puerto Rico are unevenly located across the island, favoring the San Juan Metropolitan Area [20]. This uneven distribution of healthcare resources presents considerable difficulties in accessing consistent and timely cancer treatment in patients in areas distant from the San Juan Metropolitan Area. By analyzing treatment disruption across these defined health regions, this study aims to highlight how geographic and infrastructural disparities impact cancer patients’ ability to maintain continuous care, especially in the aftermath of natural disasters.

To comprehensively assess the impact of Hurricanes Irma and María on cancer care in Puerto Rico, the present analysis focused on cancer patients and their reported experiences of treatment disruption. While this focus provides valuable insights, this manuscript does not integrate qualitative data obtained from key informants, including healthcare providers, about their understanding of barriers and facilitators related to cancer care disruption. This omission may limit the understanding of how system-level decisions and operational challenges influenced treatment disruptions. Physicians’ practices during the crisis, which were affected by hospital closures, power outages, and resource shortages, are crucial for contextualizing the nature of these disruptions. The treatment disruptions experienced by patients were largely due to systemic and infrastructural failures rather than individual medical decisions. Physicians were often limited by factors beyond their control, such as extended power outages and damaged medical facilities, making these disruptions more the result of external challenges than individual choices. Although this study concentrated on patient-level data, acknowledging these systemic issues is essential for a complete understanding of the disruptions. Both healthcare professionals and patients faced substantial challenges in maintaining continuous care during this period. The qualitative part of our study, which includes perspectives from physicians and other key informants offers a more complete view of the systemic issues contributing to treatment disruptions and helps bridge the gap between patient experiences and healthcare system challenges.

In collaboration with the Puerto Rico Central Cancer Registry (PRCCR), we conducted a multilevel mixed-method study to identify factors leading to the disruption or continuation of cancer care in Puerto Rico among breast and colorectal cancer patients diagnosed within six months prior to the hurricanes. In May and June 2019, we conducted key informant interviews and focus groups to explore the barriers and facilitators related to the disruption and continuation of cancer care in the aftermath of the hurricanes. These qualitative methods aimed to capture a broad range of perspectives and identify key issues affecting cancer patients. For the present analysis, we use quantitative data from a cross-sectional survey of breast and colorectal cancer patients who were undergoing active treatment to evaluate the impact of residence region on cancer treatment disruption following Hurricanes Irma and María in Puerto Rico.

## 2. Materials and Methods

### 2.1. Study Design and Inclusion Criteria

This study analyzes data collected to identify barriers, facilitators, and factors associated with the disruption and continuation of cancer care in the aftermath of Hurricanes Irma and María in Puerto Rico. From December 2019 to March 2021, we conducted a cross-sectional telephone survey to evaluate factors associated with cancer treatment disruption among breast and colorectal cancer patients following the hurricanes. 

Eligible participants were patients diagnosed with breast or colorectal cancer within six months before September 2017, aged 40 years or older at the time of diagnosis, who passed a Mini-Mental State test, and who were living and receiving treatment in the archipelago at the time of the hurricanes. 

### 2.2. Recruitment 

**Figure 1:**
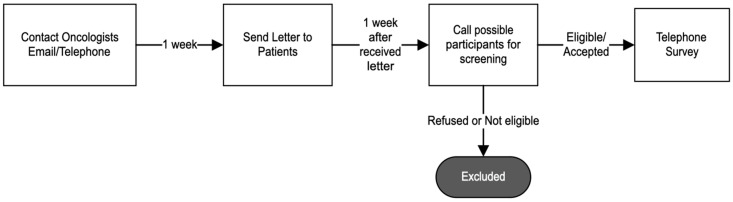
Case ascertainment protocol for population research to select participants from PRCCR data.

In collaboration with the PRCCR, one of the oldest population-based cancer registries in the world, a case ascertainment protocol was created to identify and recruit patients reported to the cancer registry (Figure 1). The list of possible participants who were diagnosed between 1 March 2017, and 31 August 2017, was provided by the PRCCR and included diagnoses from all of Puerto Rico. Participants identified using the PRCCR database were contacted by oncologists via email or telephone. With permission from their physicians, the possible participants were contacted via mail to invite them to participate and later through a phone call for eligibility screening. The telephone survey was performed between December 2019 and March 2021. We contacted a total of 398 potential participants. Of these, 73 declined to participate. Additionally, 35 participants had completed their treatment or diagnosis prior to the hurricanes. Another 23 individuals did not pass the Mini-Mental State examination. We also excluded 10 participants who did not reside in Puerto Rico either before or during the hurricanes. Furthermore, 8 participants were receiving treatment in the U.S.A. following the hurricanes, and 8 others were excluded for various other reasons. Among those contacted, 241 patients (140 with breast cancer and 101 with colorectal cancer) met the eligibility criteria for participation. Participants completed a computer-assisted telephone interview conducted by trained interviewers in Spanish, their native language, and received a financial incentive of $20 for their participation.

### 2.3. Statistical Analysis

*Dependent variable:* The primary study outcome, cancer treatment disruption, was dichotomized (yes/no) based on any pause in surgery, chemotherapy, radiotherapy, and/or oral treatment due to the hurricanes and was analyzed in comparison to continued treatment (no reported pause).

*Independent variable:* The main independent variable analyzed was the residence region, categorized using the Puerto Rico Health Department Regions (see Table 1). The San Juan Metropolitan Area (SJMA) was used as the reference region for the analysis due to its high concentration of healthcare professionals and availability of healthcare services relative to other regions. Accessibility to some healthcare services and cancer-related specialized treatment may not be available outside the SJMA, with patients from distant regions having to travel over two hours for services located in the SJMA. 

*Covariates:* Sociodemographic and clinical characteristics and hurricane-related variables were analyzed. Frequency distributions overall and by cancer treatment disruption were used to describe the participants. *p*-values were calculated using the *t*-test for continuous variables and the chi-square test for categorical variables to compare characteristics between participants who reported treatment disruption and those who did not. 

Statistical significance was set at *p* < 0.05. For bivariate analysis (Table 2, Table 3 and Table 4), we employed the *t*-test for continuous, the χ^2^-test for binary/categorical variables, and Fisher’s exact test for categorical variables having cells in the contingency table with less than 5 observations. Poisson regression models with robust variance errors were used to evaluate the association between region of residence and cancer treatment disruption (yes/no). Comparisons were made between the SJMA and the rest of the island (outside the SJMA), as well as between the SJMA and the other six Puerto Rico Health Department regions: Arecibo, Bayamón, Caguas, Fajardo, Mayagüez, and Ponce. The Akaike Information Criterion (AIC) and Bayesian Information Criterion (BIC) were the metrics used to evaluate the model fitness. They help compare and choose the best-fitting model among a set of candidate models, balancing model fit and complexity. We present unadjusted prevalence ratios of cancer treatment disruption by region of residence and prevalence ratios adjusted for age and marital status.

## 3. Results

Of the participants, 47% were between 40 and 60 years old, 63% were married, and 81% were women. More than half (59%) reported cancer treatment disruption; among them, half (50%) experienced disruptions lasting more than 30 days, with 14% of these lasting more than 90 days. Table 2 presents sociodemographic characteristics overall and by cancer treatment disruption. Other variables such as sex, caregiver status, education level, annual income, government benefits, socioeconomic status, employment, and transportation did not show an association with treatment disruption. A significant association was found for age, marital status, and health insurance, suggesting potential associations between these variables and cancer treatment disruption (*p* = 0.05).

**Table 2:** Sociodemographic characteristics by cancer treatment disruption.

	Cancer Treatment Disruption			
	No (99) n (%)	Yes (142) n (%)	Total (241) n (%)	*p*-Value
**Sex**				0.86
Male	19 (19.2)	26 (18.3)	45 (18.7)	
Female	80 (80.8)	116 (81.7)	196 (81.3)	
Age (mean ± SD)	62.6 (10.6)	59.4 (9.9)	60.7 (10.3)	**0.01**
Marital Status				0.05
Unmarried	29 (29.3)	59 (41.5)	88 (36.5)	
Married	70 (70.7)	83 (58.5)	153 (63.5)	
Caregiver				0.89
No	74 (74.7)	105 (73.9)	179 (74.3)	
Yes	25 (25.3)	37 (26.1)	62 (25.7)	
Education Level				0.65
≤12 years	37 (37.4)	49 (34.5)	86 (35.7)	
>12 years	62 (62.6)	93 (65.5)	155 (64.3)	
Annual Income ^1^				0.46
<$15,000	25 (25.5)	39 (27.5)	64 (26.7)	
≥$15,000	73 (74.5)	103 (72.5)	176 (73.3)	
Government Benefits ^2^				0.13
No	72 (72.7)	90 (63.4)	162 (67.2)	
Yes	27 (27.3)	52 (36.6)	79 (32.8)	
Health Insurance				**0.05**
Medicare/Medicaid	35 (35.4)	72 (50.7)	107 (44.4)	
Private	44 (44.4)	50 (35.2)	94 (39.0)	
Medicare	19 (19.2)	16 (11.3)	35 (14.5)	
None	1 (1.0)	4 (2.8)	5 (2.1)	
SVI ^3^				0.14
High	17 (17.2)	30 (21.1)	47 (19.5)	
Medium	28 (28.3)	53 (37.3)	81 (33.6)	
Low	54 (54.5)	59 (41.5)	113 (46.9)	
Employment ^4^				0.19
No	76 (76.8)	98 (69.0)	174 (72.2)	
Yes	23 (23.2)	44 (31.0)	67 (27.8)	
Transportation				0.76
Own	34 (34.3)	43 (30.3)	77 (32.0)	
Family	61 (61.6)	94 (66.2)	155 (64.3)	
Other	4 (4.0)	5 (3.5)	9 (3.7)	

*p*-values were calculated using the *t*-test for continuous age, the χ^2^ test for binary/categorical variables, and Fisher’s exact test for binary/categorical variables with cells having less than 5 observations (health insurance and transportation). ^1^ One person did not answer the annual income question, n = 240. ^2^ Government benefits: Are you a participant in any government assistance program? For example, the Supplemental Nutrition Assistance Program (SNAP), Temporary Assistance for Needy Families (TANF), Section 8, etc., excluding health program assistance (Medicaid). ^3^ Social Vulnerability Index (SVI). ^4^ Employment: “Yes” includes full-time or part-time and self-employed; “No” includes unemployed and looking for a job, retired, disabled, not working and not looking for a job, and homemaker. Bold values represent *p*-values < 0.05.

**Figure 2:**
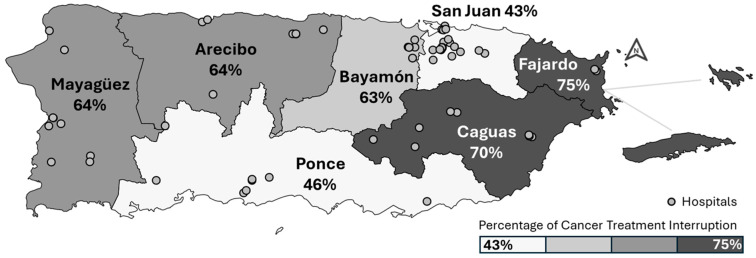
Cancer treatment disruption and location of hospitals in the Puerto Rico Department of Health Regions.

Figure 2 shows the disruption of cancer treatment across the health regions of Puerto Rico. The SJMA region had the lowest percentage of treatment disruption, with 43% of participants reporting issues, followed by the Ponce region at 46%. In contrast, the Fajardo and Caguas regions experienced the highest levels of disruption, with 75% and 70% of residents, respectively, reporting treatment disruption.

Table 3 shows cancer type and stage by cancer treatment disruption. Fifty-eight percent of participants had breast cancer, while 42% had colorectal cancer. Half of the participants (49%) who reported treatment disruptions were diagnosed with regional or distant-stage cancer. There is a statistical difference in stage when comparing persons with and without treatment disruption (*p* = 0.02).

**Table 3:** Cancer type and stage by treatment disruption.

	Cancer Treatment Disruption			
	No (n = 99) n (%)	Yes (n = 142) n (%)	Total (241) n (%)	*p*-Value
Cancer Type				0.15
Breast	63 (63.6)	77 (54.2)	140 (58.1)	
Colorectal	36 (36.4)	65 (45.8)	101 (41.9)	
Stage				**0.02**
Localized	61 (61.6)	73 (51.4)	134 (55.6)	
Regional	38 (38.4)	61(43.0)	99 (41.1)	
Distant	0 (0)	8 (5.6)	8 (3.3)	

*p*-values were calculated using the χ^2^-test for cancer type and Fisher’s exact test for stage. The bold value represent a *p*-value < 0.05.

Table 4 shows the disruption, stage, and delay by treatment type. Out of the 241 participants, 232 (96%) underwent surgical treatment, with 30 (13%) reporting disruption. Of these, eight (27%) were at the regional or distant stage, excluding two observations with missing data. Additionally, twelve (43%) reported a delay of 30–60 days, and nine (32%) reported a delay greater than 60 days. 

Chemotherapy was reported by 153 (64%) participants, with 83 (54%) reporting disruption. Of these, 54 (65%) were at the regional or distant stage, excluding 31 observations with missing data. Furthermore, 27 (52%) reported a delay of 30–60 days, and 15 (29%) reported a delay greater than 60 days.

Radiotherapy was reported by 120 (50%) participants, with 42 (35%) reporting disruption. Of these, 17 (41%) were at the regional or distant stage, excluding 20 observations with missing data. In addition, nine (41%) reported a delay of 30–60 days, and four (18%) reported a delay greater than 60 days.

Oral treatment was reported by fifty-nine (25%) participants, with six (10%) reporting disruption. Of these, two (33%) were at the regional stage; the only participant who reported a delay indicated it was for greater than 60 days.

**Table 4:** Stage and delay of disruption by treatment type.

Treatment, n (%)	Disruption, n (%)		
Surgery, 232 (96.3)	30 (12.9)		
Stage		Delay (days)	(2 missing)
Localized	22 (73.3)	<30	7 (25.0)
Regional	7 (23.3)	30–60	12 (42.9)
Distant	1 (3.3)	>60	9 (32.1)
Chemotherapy, 153 (63.5)	83 (54.3)		
Stage		Delay (days)	(31 missing)
Localized	29 (34.9)	<30	10 (19.2)
Regional	48 (57.8)	30–60	27 (51.9)
Distant	6 (7.2)	>60	15 (28.9)
Radiotherapy, 120 (49.8)	42 (35.0)		
Stage		Delay (days)	(20 missing)
Localized	25 (59.5)	<30	9 (40.9)
Regional	15 (35.7)	30–60	9 (40.9)
Distant	2 (4.8)	>60	4 (18.2)
Oral, (59, 24.5)	6 (10.2)		
Stage		Delay (days)	(5 missing)
Localized	4 (66.7)	>60	1 (100)
Regional	2 (33.3)		
Distant	0 (0)		

Table 5 presents hurricane-related variables by cancer treatment disruption. Most participants reported high support (91%), lower preparedness (72%), an absence of traumatic (76%) and non-traumatic (58%) stressors, normal levels of psychological distress (64%), and a lack of medicine (82%). A significant difference in treatment disruption was found between participants who reported high preparedness and those who did not: 23% of participants in the group experiencing cancer treatment disruption reported high preparedness, whereas 35% of those in the group without disruption reported high preparedness (*p* = 0.04). A significant difference in treatment disruption was also found between participants who reported a lack of medicine and those who did not: 23% of participants in the group experiencing cancer treatment disruption reported a lack of medicine, whereas 12% of those in the group without disruption reported a lack of medicine (*p* = 0.04).

The best-fitting models based on AIC and BIC were the unadjusted models. Models adjusted for age and marital status, which were borderline statistically significant regarding cancer treatment disruption in the bivariate analysis, showed very similar AIC and BIC values. Adjustments for health insurance, high preparedness, or lack of medicine, which were statistically significant in the bivariate analysis, did not change the estimates and resulted in less well-fitting models compared to the unadjusted models.

Table 6 shows the prevalence ratios of cancer treatment disruption by residence region. Models adjusted for age and marital status revealed that cancer treatment disruption was 48% more prevalent among cancer patients residing outside the SJMA (PR = 1.48, 95% CI: 1.06–2.07) compared to those residing within the SJMA. Additionally, in models adjusted for age and marital status, cancer patients in Arecibo (PR = 1.51, 95% CI: 1.03–2.21), Bayamón (PR = 1.50, 95% CI: 1.01–2.23), Caguas (PR = 1.60, 95% CI: 1.09–2.34), and Mayagüez (PR = 1.52, 95% CI: 1.02–2.26) exhibited higher prevalence of cancer treatment disruption compared to those residing in the SJMA.

## 4. Discussion

Our study provides insight into the profound impact of Hurricanes Irma and María on healthcare delivery in Puerto Rico, with a particular focus on the association between residence region and cancer treatment disruption. We observed a significant disparity in treatment disruption, with individuals outside the SJMA more likely to experience disruptions in the disasters’ aftermath, independent of sex, insurance status, education, and income. Specifically, residents outside the SJMA faced a ~50% higher prevalence of cancer treatment disruption compared to those within the SJMA. 

In our study, the southern coastal Ponce region showed a similar prevalence of cancer treatment disruption to the SJMA. Although this region includes 15 individual municipalities, it is noteworthy that 42% of our participants from this region hailed from the municipality of Ponce itself. Ponce is a major city in Puerto Rico and the most populated city outside the SJMA, equipped with several long-standing chemotherapy and radiotherapy centers [14]. Despite a small sample size, the Fajardo region exhibited the highest estimate of treatment disruption, though not statistically significant, indicating potential vulnerability. This result is notable even though we did not survey participants from the island municipalities of Vieques and Culebra, which are part of the Fajardo region. Future research should aim to include a larger sample from this region, specifically incorporating participants from Vieques and Culebra. These island municipalities, isolated from the mainland and facing limited transportation (ferry) access post-hurricanes, lack local cancer care centers. As a result, residents must travel to the mainland for treatment, which exacerbates treatment disruptions. Addressing these factors in future studies will be crucial for understanding and mitigating the impact of treatment disruptions in this vulnerable region.

Participants who did not report treatment disruption were more likely to report high disaster preparedness than those who reported treatment disruption. This finding highlights the connection between individual perceptions of preparedness, access to resources, and overall resilience. In a globally interconnected and rapidly changing environment, the interplay between personal concerns, exposures, and preparedness transcends mere correlation, revealing deeper causal dynamics that influence how individuals manage and respond to risks [21]. This emphasizes the importance of integrating disaster preparedness recommendations into cancer care management plans, ensuring that patients have access to essential medications, resources, and support services before, during, and after disasters. Collaboration across various levels of government is crucial to identify cancer patients undergoing treatment and to devise interventions or alternative strategies to reduce treatment disruptions [22].

Our analysis of treatment disruptions by type of cancer treatment reveals notable differences in the impact of Hurricanes Irma and María on various cancer treatments. Disruptions were most prevalent for chemotherapy, with more than half of those undergoing this treatment reporting interruptions. This is particularly concerning given that almost two-thirds of those who experienced disruptions in chemotherapy were diagnosed with regional or distant-stage cancers, which typically require more intensive and frequent treatments. The high prevalence of disruption in chemotherapy may be attributed to the complexity and scheduling demands of this treatment modality, which makes it more vulnerable to service disruptions. 

Surgical treatments also showed a significant disruption rate, with 13% of those who underwent surgery reporting delays. More than forty percent of these delays were between 30 and 60 days, and almost a third faced delays greater than 60 days. This is critical, as timely surgical intervention is often crucial for managing advanced stages of cancer. The delays in surgical treatments could have substantial implications for patient outcomes, particularly for those with regional or distant-stage cancers. Among participants with regional or distant-stage cancers, more than half reported a delay greater than 60 days, and eighty-six percent reported a delay greater than 30 days. 

More than a third of those undergoing radiotherapy reported disruptions, with fewer experiencing significant delays. This might reflect the more flexible scheduling and potentially less intensive nature of radiotherapy compared to chemotherapy. Oral treatments had the lowest disruption rate, with ten percent reporting interruptions. This suggests that oral therapies may offer a more resilient approach to cancer management during emergencies, as they are less dependent on specialized infrastructure and can be more easily administered outside of clinical settings. These findings emphasize the need for tailored disaster preparedness strategies that account for the specific vulnerabilities of different cancer treatment types. Ensuring the continuity of chemotherapy and surgical treatments requires focused efforts to maintain access to essential services and minimize delays. Additionally, the resilience of oral therapies under disaster conditions highlights their potential utility in disaster preparedness and response planning for cancer care.

Due to the critical nature of the surgery and its scheduling as a single event, rescheduling data for this treatment is nearly complete, with only two missing values out of 232. In contrast, rescheduling data for other treatments proved more challenging to recall, resulting in higher rates of missing observations: thirty-one out of eighty-three for chemotherapy, twenty out of forty-two for radiotherapy, and five out of six for oral treatment. Thus, caution must be exercised when interpreting disruptions by cancer treatment type.

The significant association between a lack of medicine and treatment disruption underscores the critical role of medication supply chains in ensuring continuity of care during emergencies. Addressing these barriers requires a coordinated effort involving healthcare providers, policymakers, and community stakeholders to strengthen supply chain resilience, enhance access to essential medications for all cancer patients, and optimize adherence through effective collaboration with patients and their care teams [23].

As reported in a previous publication from this project, difficulty in communicating with healthcare providers and facilities was undoubtedly one of the most pressing barriers during this disaster [24]. Enhancing the use of technology might help to create better continuity in cancer care and empower individuals by providing them with more control over how they access and utilize support services, enabling them to tailor their experiences to better meet their needs. This approach can assist cancer patients in achieving better adherence to treatment amid the challenges they confront, which is crucial for their well-being and treatment outcomes [25].

Despite sound analysis and significant results, our study is subject to some limitations, including a small sample size and reliance on self-reported data, which may be influenced by recall bias. The sample size of 241 eligible participants, while substantial, may still limit the generalizability of the study’s findings. Despite the use of a well-established cancer registry for recruitment, the final sample may not fully represent the diverse cancer patient population in Puerto Rico. The relatively small sample size, particularly in specific regions like Fajardo, limits the power of this study to detect significant differences and may skew the results. Larger and more geographically representative samples would enhance the robustness of the findings and allow for more detailed subgroup analyses. 

Our study focused exclusively on patients with breast and colorectal cancers. While these are prevalent cancer types and their inclusion provides important insights, the findings may not be generalizable to patients with other types of cancer. Different cancers have distinct treatment regimens, follow-up requirements, and potentially varying levels of treatment disruption. For example, patients with hematologic cancers or those requiring frequent inpatient care might experience different challenges compared to those with breast or colorectal cancers. 

Participants were offered a $20 incentive for their involvement in this study. Even though it is unlikely, given the small amount, the incentive might have introduced a selection bias, potentially skewing the sample towards individuals of lower socioeconomic status, which could also influence geographic location and impact this study’s findings. Additionally, data collection via telephone surveys introduces the possibility of non-response bias. Compounding these challenges is the fact that a significant portion of participant recruitment occurred during the COVID-19 pandemic. Our study did not explore rural–urban differences in treatment disruption due to the methodological challenges in describing rural–urban regions on the island [26]; instead, we focused on regional differences. 

Despite these limitations, this study has notable strengths, including the development and utilization of a case ascertainment protocol in collaboration with the PRCCR for identifying cancer patients. This supports future population-based studies in collaboration with the cancer registry, enhancing the research team’s ability to capture comprehensive and geographically diverse data due to the centralized nature of the PRCCR. Leveraging a population-based cancer registry for participant selection helps mirror the wider population, bolstering this study’s validity and generalizability. 

## 5. Conclusions

Our study highlights the critical importance of geographic location in determining access to healthcare resources, particularly in disaster-prone areas like Puerto Rico and in the aftermath of a disaster. Addressing regional disparities in cancer care resilience is imperative for ensuring equitable access to treatments and services. The higher prevalence of treatment disruption outside the SJMA underscores the challenges faced by vulnerable populations in rural and underserved areas. Integrating these insights into disaster preparedness strategies is essential for enhancing healthcare resilience and mitigating disparities in access to cancer care. Further research is needed to explore the specific impacts of natural disasters on cancer care access and outcomes, particularly among vulnerable populations.

To address the limitations identified in our study and build on our findings, several areas for future research are crucial. First, larger and more geographically representative samples are needed to validate our findings and improve the generalizability of results, particularly in less-studied regions like Fajardo and the island municipalities of Vieques and Culebra. Second, research should expand to include a broader range of cancer types beyond breast and colorectal cancers, as different cancers may present unique challenges in treatment continuity during disasters. Third, future studies should explore the effectiveness of various disaster preparedness strategies specifically tailored for cancer care, including technological interventions and communication improvements, to mitigate treatment disruptions. Additionally, addressing limitations such as recall bias through electronic health records and other alternative data collection methods will be essential. Ensuring a more diverse participant pool will enhance the robustness of future research. 

In conclusion, our study emphasizes the urgent need for equitable access to cancer care resources and the integration of disaster preparedness into healthcare management plans. By addressing access barriers and disparities across regions, we can safeguard the continuity of cancer care, particularly in vulnerable areas. Collaborative efforts across sectors are crucial to strengthening healthcare resilience, promoting health equity, and minimizing the impact of future disasters on cancer care access and outcomes.

## Figures and Tables

**Table 1:** Municipalities in the Puerto Rico Health Department Regions.

Region						
Arecibo	Bayamón	Caguas	Fajardo	Mayagüez	Metro	Ponce
Arecibo	Barranquitas	Aguas Buenas	Ceiba	Aguada	Canóvanas	Adjuntas
Barceloneta	Bayamón	Aibonito	Culebra	Aguadilla	Carolina	Arroyo
Camuy	Cataño	Caguas	Fajardo	Añasco	Guaynabo	Coamo
Ciales	Comerío	Cayey	Luquillo	Cabo Rojo	Loíza	Guánica
Florida	Corozal	Cidra	Río Grande	Hormigueros	San Juan	Guayama
Hatillo	Dorado	Gurabo	Vieques	Isabela	Trujillo Alto	Guayanilla
Lares	Naranjito	Humacao		Lajas		Jayuya
Manatí	Orocovis	Juncos		Las Marías		Juana Díaz
Morovis	Toa Alta	Las Piedras		Maricao		Patillas
Quebradillas	Toa Baja	Maunabo		Mayagüez		Peñuelas
Utuado	Vega Alta	Naguabo		Moca		Ponce
Vega Baja		San Lorenzo		Rincón		Salinas
		Yabucoa		Sabana Grande		Santa Isabel
				San Germán		Villalba
				San Sebastián		Yauco

**Table 5:** Hurricane-related variables by cancer treatment disruption.

	Cancer Treatment Disruption			
	No (99) n (%)	Yes (142) n (%)	Total (241) n (%)	*p*-Value
Social Support				0.27
Low	11 (11.1)	10 (7.0)	21 (8.7)	
High	88 (88.8)	132 (93.0)	220 (91.3)	
High Preparedness				**0.04**
No	64 (64.6)	109 (76.8)	173 (71.8)	
Yes	35 (35.4)	33 (23.2)	68 (28.2)	
Traumatic Stressors				0.82
Absent	74 (74.7)	108 (76.1)	182 (75.5)	
Present	25 (25.3)	34 (23.9)	59 (24.5)	
Non-Traumatic Stressors ^1^				0.64
Absent	57 (59.4)	80 (56.3)	137 (57.6)	
Present	39 (40.6)	62 (43.7)	101 (42.4)	
Psychological Distress				0.20
Normal	68 (68.7)	86 (60.6)	154 (63.9)	
High	31 (31.3)	56 (39.4)	87 (36.1)	
Lack of Medicine ^2^				**0.04**
No	86 (87.8)	110 (77.5)	196 (81.7)	
Yes	12 (12.2)	32 (22.5)	44 (18.3)	

*p*-values were calculated using the χ^2^-test for binary/categorical variables. ^1^ Three persons did not answer the non-traumatic stressors question, n = 238. ^2^ One person did not answer the lack of medicine question, n = 240. The bold value represent a *p*-value < 0.05.

**Table 6:** Prevalence ratios of cancer treatment disruption by residence region.

	Unadjusted PR (95% CI)	Adjusted PR (95% CI) ^1^
Model 1		
SJMA	1.0	1.0
Outside the SJMA	**1.48 (1.04, 2.10)**	**1.48 (1.06, 2.07)**
Model 2		
San Juan	1.0	1.0
Arecibo	**1.50 (1.01, 2.23)**	**1.51 (1.03, 2.21)**
Bayamón	1.48 (0.98, 2.21)	**1.50 (1.01, 2.23)**
Caguas	**1.65 (1.11, 2.45)**	**1.60 (1.09, 2.34)**
Fajardo	1.76 (0.91, 3.40)	1.84 (0.93, 3.65)
Mayagüez	**1.51 (1.00, 2.26)**	**1.52 (1.02, 2.26)**
Ponce	1.08 (0.62, 1.86)	1.10 (0.64, 1.86)

^1^ Adjusted for age and marital status; PR = Prevalence ratio; PRs in bold are statistically significant (*p* < 0.05).

## Data Availability

Data is unavailable due to privacy and ethical restrictions.
